# Detection of fetal structural abnormalities at 20 and 30 weeks: a retrospective study of prenatal ultrasound screening

**DOI:** 10.1007/s10396-025-01609-1

**Published:** 2025-12-13

**Authors:** Yuki Kamihara, Katsusuke Ozawa, Kohei Ogawa, Jin Muromoto, Rika Sugibayashi, Seiji Wada, Haruhiko Sago

**Affiliations:** Center for Maternal-Fetal, Neonatal and Reproductive Medicine, National Center for Child Health and Development, 2-10-1 Okura Setagaya-ku, Tokyo, 157-8535 Japan

**Keywords:** Congenital abnormalities, Fetal diseases, Prenatal diagnosis, Third trimester, Ultrasound screening

## Abstract

**Purpose:**

To evaluate the role of ultrasound screening for fetal structural abnormalities at 20 and 30 weeks’ gestation.

**Methods:**

This retrospective study included women with singleton pregnancies who underwent routine ultrasound screening at our hospital between May 2014 and April 2021. Participants received two ultrasound screening examinations: at 20 and 30 weeks’ gestation. We evaluated the frequency and details of fetal structural abnormalities identified during each examination.

**Results:**

A total of 10,560 pregnant women underwent ultrasound screening at 20 and 30 weeks’ gestation and delivered at our hospital. Structural abnormalities were observed in 142 (1.3%) women; of note, screening examinations at 20 and 30 weeks detected 42.3% and 23.9% of cases, respectively. Routine point-of-care ultrasound examinations performed between 20 and 30 weeks and beyond 30 weeks detected 8.5% and 6.3% of cases, respectively, with 19.0% of cases identified after birth. Cleft lip and palate and ventricular septal defects (VSDs) were the most common abnormalities detected at the 20-week ultrasound screening. At the 30-week ultrasound screening, in addition to hydronephrosis, VSD, and vascular rings, which were also detected at the 20-week ultrasound screening, diaphragmatic hernia, ovarian cysts, achondroplasia, duodenal atresia, and meconium peritonitis, which could be apparent later in pregnancy, were detected.

**Conclusions:**

The 20-week ultrasound screening revealed common fetal structural abnormalities, and the 30-week ultrasound screening detected fetal structural abnormalities that failed to be detected during the 20-week ultrasound screening and became apparent later in pregnancy. Adding a 30-week ultrasound screening provides enhanced diagnostic details in comparison to 20-week screening.

## Introduction

Ultrasound screening for fetal structural abnormalities can improve prenatal diagnoses and postnatal management of congenital diseases and even allow for fetal therapy in some cases. It also allows couples to choose to terminate their pregnancies in cases of a poor prognosis [1, 2]. Fetal ultrasound screening is now commonly used because of the availability of ultrasound equipment. Guidelines from the United States, the United Kingdom, and International Society of Ultrasound in Obstetrics and Gynecology (ISUOG) suggest that it should be performed once at approximately 20 weeks of pregnancy [2–4]. This timeframe enables evaluation of the detailed anatomy of the fetus. Detecting congenital diseases at an early stage provides ample time for preparation before birth or the option for couples to terminate the pregnancy [2].

Recently, researchers have studied the benefits of using fetal ultrasound screening during the third trimester in addition to the standard 20 weeks’ gestation period [5]. Studies have evaluated both incidental fetal structural abnormalities found during ultrasound examinations aimed at monitoring fetal development and position in the third trimester, as well as the effectiveness of ultrasound in detecting fetal abnormalities during this period. In Japan, ultrasound examinations are frequently performed not only to evaluate fetal structural screening but also to assess estimated fetal weight and amniotic fluid volume. The advantages of frequent ultrasound examinations for detecting severe fetal growth restriction (FGR) have been reported previously [6, 7]. Japanese guidelines recommend that ultrasound screening for fetal abnormalities be performed three times during pregnancy: at 10–13 weeks, 18–20 weeks, and 28–31 weeks, as necessary [6]. However, insufficient scientific evidence supports the value of an ultrasound scan in the third trimester, in addition to a fetal ultrasound scan in the second trimester.

In the present study, we examined the frequency and categorization of fetal structural abnormalities detected at ultrasound examinations performed at different time points during pregnancy. We then evaluated the role of a fetal ultrasound screening examination at 30 weeks’ gestation in addition to that at 20 weeks’ gestation.

## Methods

This retrospective study was conducted at a tertiary-care perinatal center in Japan. The initial study population was composed of women with singleton pregnancies who underwent routine 20-week ultrasound screening at our institution between May 2014 and April 2021.

From this population, we excluded the following cases to ensure that the participants had completed the entire institutional screening protocol: (1) women who were transferred to other facilities after the 20-week scan, (2) those who delivered before undergoing the 30-week scan (including preterm births), and (3) those who experienced intrauterine fetal demise prior to the 30-week scan.

**Fig. 1:**
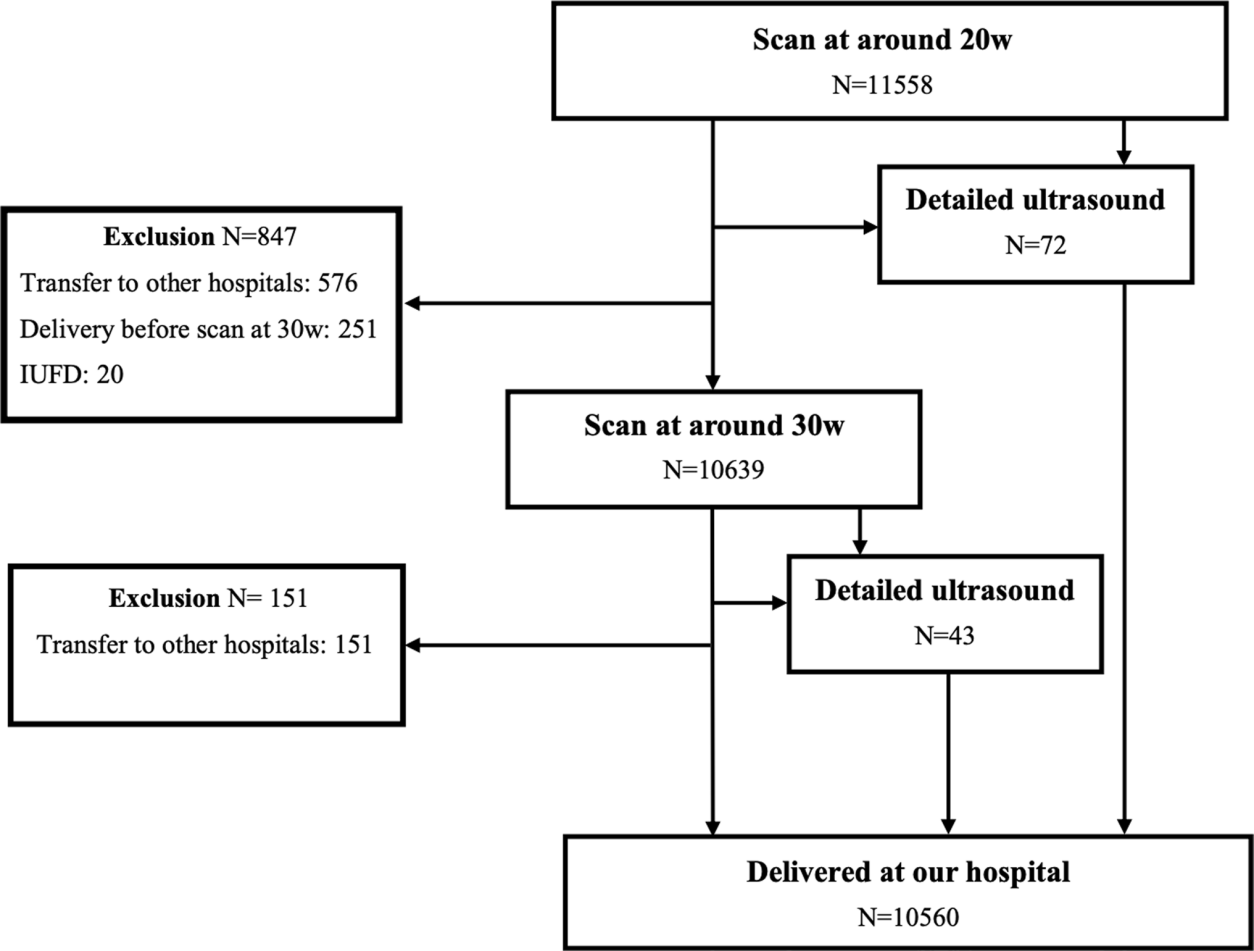
A flow chart showing the study population. In total, 11,558 people were scanned at around 20 weeks, with 847 later excluded. A total of 10,639 were scanned at around 30 weeks, with 151 transferred, leaving 10,560 mothers ultimately giving birth in our hospital. *IUFD* intrauterine fetal demise, *N* number, *W* weeks’ gestation

Women who were transferred to another facility after completing the 30-week ultrasound screening were excluded from the final analysis. Eligibility and exclusion criteria were determined based on a retrospective review of the medical records. The final cohort consisted of women who underwent both 20-week and 30-week ultrasound screenings and delivered at our institution (Fig. 1).

In addition to routine antenatal check-ups, fetal ultrasound screening to assess fetal abnormalities was performed twice at our hospital. The first screening ultrasound was performed at 18–21 weeks’ gestation, and the second was at 28–31 weeks’ gestation. The screening examinations were performed following ISUOG-based protocols and included an evaluation of the head (the cavum septum pellucidum, lateral cerebral ventricles, midline falx, cerebellum, and cisterna magna), face (eyes, upper lip), lungs, heart (four-chamber view, transverse cardiac diameter, atrioventricular valve, pulmonary veins, three-vessel view, three-vessel and trachea view, left and right ventricular outflow tracts), stomach (presence, size, and situs), kidneys, urinary bladder, umbilical cord (insertion site into the fetal abdomen and umbilical cord vessel number), limbs, and spine [2, 4, 8]. The ultrasound examinations, which lasted around 30 min, were carried out by obstetricians under board-certified fellows of the Japan Society of Ultrasonics in Medicine (JSUM) or JSUM-registered medical sonographers. All examiners had completed institutional training in prenatal structural screening, and consistent protocols were followed. The ultrasound equipment used was the Aplio 500 ultrasound scanner (Canon Medical Systems, Tochigi, Japan) or Voluson E8 or E10 (GE HealthCare Japan, Tokyo, Japan). When abnormal findings were detected, a further detailed ultrasound examination was performed by fetal medicine specialists to evaluate the presence of structural abnormalities.

During routine antenatal check-ups (every 4 weeks between weeks 16 and 23 and every 2 weeks from weeks 24 to 37), routine point-of-care ultrasound focusing on fetal growth and position was performed [6]. An Aplio 300 ultrasound scanner (Canon Medical Systems) or Voluson E8 (GE HealthCare Japan) was used. Obstetricians at the outpatient clinic performed routine point-of-care ultrasound. Although these routine point-of-care ultrasound examinations were part of routine clinical care and not primarily intended for structural screening, fetal abnormalities were sometimes identified during these evaluations.

Structural abnormalities were identified based on retrospective review of medical records and stored ultrasound images. All cases with confirmed fetal structural abnormalities were classified according to the timing of detection into five categories: (1) at the 20-week screening, (2) during routine point-of-care ultrasound between the 20- and 30-week screenings, (3) at the 30-week screening, (4) during routine point-of-care ultrasound after the 30-week screening, and (5) after birth.

For each confirmed case, the specific type of structural abnormality and time of detection were recorded. Based on this dataset, a comprehensive list of all structural abnormalities and the timing of their detection was compiled.

From this list, the abnormalities most frequently observed during the 20-week screening, 30-week screening, and after birth were systematically extracted and ranked to provide a comparative summary of the most common conditions detected at each time point. To confirm the diagnosis, all records of fetal abnormalities were reviewed using reference images. If an abnormality was detected at 20 weeks’ gestation, no additional screening test was performed at 30 weeks. The postnatal period for detecting structural abnormalities was limited to 1 week after birth as the neonates were discharged from the hospital at that time. Abnormal fetal findings did not include non-structural abnormalities, such as FGR, amniotic fluid abnormalities, and placental–umbilical cord abnormalities.

## Results

A total of 10,560 pregnant women underwent the ultrasound screening protocol and delivered at our hospital (Fig. 1). Their characteristics are presented in Table 1. Their mean age was 36.2 years, and two-thirds of them were over 35 years old.

**Table 1:** Characteristics of pregnant women (*n* = 10,560)

Characteristics	Mean (SD) or *N* (%)
Age (years)	36.2 (4.5)
≤ 34	3601 (34)
35–39	4262 (40)
40–44	2519 (24)
≥ 45	178 (2)
Japanese	10,402 (99)
Height (cm)^a^	159.4 (11.3)
Body weight at booking (kg)^b^	52.7 (8.0)
BMI at booking (kg/m^2^)^c^	20.7 (2.9)
Nulliparous	6156 (58)
Smoking	
Current smoker	63 (0.6)
Ex-smoker	557 (5.3)
Never smoker	9940 (94.1)
Preexisting diabetes mellitus	33 (0.3)
In vitro fertilization	2650 (25)

Data are expressed as mean (standard deviation) or number (%)

*BMI* body mass index, *N* number, *SD* standard deviation

^a^There are 432 missing data

^b^There are 742 missing data

^c^There are 1092 missing data

**Fig. 2:**
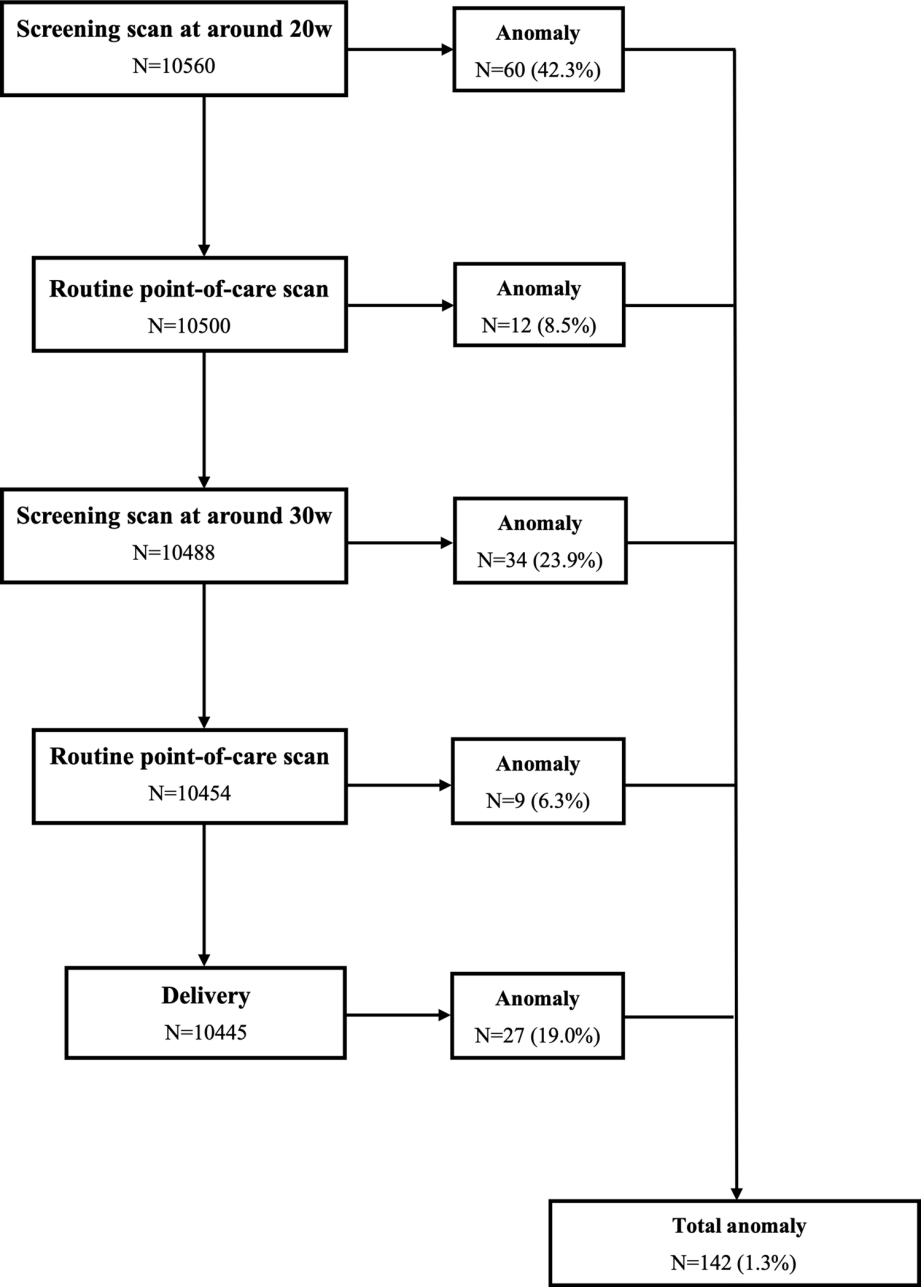
A flow chart showing the outcomes according to the timing of detection. Overall, structural abnormalities were detected in 142 cases (1.3%). *N* number, *W* weeks’ gestation

Figure 2 shows the outcomes according to the timing of detection. Structural abnormalities were detected in 142 patients (1.3%). Among them, 115 (81.0%) were identified during prenatal screening examinations, and among them, 60 (42.3%) were detected at the 20-week screening, while 34 (23.9%) were detected at the 30-week screening. Twelve (8.5%) were detected at routine point-of-care ultrasound between the 20- and 30-week screenings, and nine (6.3%) were found at routine point-of-care ultrasound after the 30-week screening. The remaining 27 (19.0%) were detected after birth.

Table 2 shows the list of congenital abnormalities detected at the 20- and 30-week ultrasound screenings and just after birth. Cleft lip was the most common disorder detected at the 20-week ultrasound screening, followed by ventricular septal defect (VSD). Pyelectasis was the most common disorder detected at the 30-week ultrasound screening, followed by VSD. Ovarian cysts and achondroplasia were also detected at the 30-week ultrasound screening.

**Table 2:** List of structural abnormalities detected at two ultrasound screenings and after birth, in order of frequency

At 20 w	*N*	At 30 w	*N*	PN	*N*
CLP	10	Pyelectasis	4	VSD	5
VSD	6	VSD	4	Anal atresia	4
Vascular ring	5	Vascular ring	4	Multiple malformations	4
Pyelectasis	5	CDH	3	Spinal lipoma	2
Multiple malformations	5	VM	2	Cervical lymphangiomas	2
VM	3	MCM	2	CDH	2
Heterotaxy syndrome	3	PA^a^	2	Others	8
MCDK	3	Ovarian cyst	2	Total	27
Others	20	Achondroplasia	2		
Total	60	Others	9		
		Total	34		

*CDH* congenital diaphragmatic hernia, *CLP* cleft lip and palate, *MCDK* multicystic dysplastic kidney, *MCM* megacisterna magna, *N* number, *PA* pulmonary atresia, *PN* postnatal, *VM* ventriculomegaly, *VSD* ventricular septal defect, *W* weeks’ gestations

^a^PA includes PA with both intact ventricular septum and VSD

Table 3 shows the details of the structural abnormalities and timing of detection. Of the 16 cases with central nervous system abnormalities, nine had ventriculomegaly. Five of the 16 cases were identified during the 20-week ultrasound screening. Among the 15 cases with facial and neck disorders, 11 were cleft lip and palate, most of which were found at the 20-week ultrasound screening.

**Table 3:** Details of structural abnormalities and the timing of detection

Abnormalities	Scan at 20 w	Between scans	Scan at 30 w	After scans	PN	Total
Central nervous system	5	3	4	1	3	16
VM	3	2	2	1	1	9
MCM^b^			2			2
Spinal lipoma					2	2
ACC		1				1
Brain tumor	1					1
Arachnoid cyst	1					1
Face and neck	10	0	2	0	3	15
CLP	10		1			11
Cervical lymphangiomas^b^			1		2	3
Cleft palate only					1	1
Thorax	4	3	4	0	2	13
CDH	1	2	3		2	8
Pleural effusion	1	1				2
BPS	2					2
PMM^b^			1			1
Cardiovascular	25	1	14	2	8	50
VSD	6		4	1	5	16
Vascular ring	5		4	1	1	11
DORV	2		1		1	4
ToF	2		1			3
Heterotaxy syndrome	3					3
PA^a^	1		2			3
PLSVC	2					2
TAPVC	1		1			2
Ebstein anomaly					1	1
TGA^b^			1			1
Truncus arteriosus	1					1
TA	1					1
Cardiac tumor	1					1
Ventricular aneurysm		1				1
Gastrointestinal	2	1	2	2	6	13
Anal atresia					4	4
Hepatomegaly^b^		1	1		1	3
Duodenal atresia^b^			1			1
Choledochal cyst	1					1
Ascites	1					1
Meconium peritonitis				1		1
Intestinal malrotation					1	1
Bowel atresia				1		1
Genitourinary	9	3	6	4	1	23
Pyelectasis	5	1	4	1		11
MCDK	3	2				5
Ovarian cyst^b^			2	1	1	4
Adrenal hemorrhage	1			1		2
PUV				1		1
Skeleton	0	0	2	0	0	2
Achondroplasia^b^			2			2
Other	5	1	0	0	4	10
Multiple malformations	5				4	9
Hydrops fetalis		1				1
Total	60	12	34	9	27	142

*ACC* agenesis of the corpus callosum, *BPS* bronchopulmonary sequestration, *CDH* congenital diaphragmatic hernia, *CLP* cleft lip and palate, *DORV* double outlet right ventricle, *MCDK* multicystic dysplastic kidney, *MCM* megacisterna magna, *PA* pulmonary atresia, *PLSVC* persistent left superior vena cava, *PMM* posterior mediastinal mass, *PN* postnatal, *PUV* posterior urethral valve, *TGA* transposition of the great arteries, *TA* tricuspid atresia, *TAPVC* total anomalous pulmonary venous connection, *ToF* tetralogy of Fallot, *VM* ventriculomegaly, *VSD* ventricular septal defect, *W* weeks’ gestations

^a^PA includes both pulmonary atresia with intact ventricular septum and PA with ventricular septal defect

^b^Detected at the 30-week screening but not identified at 20 weeks

Among the 13 cases with thoracic abnormalities, congenital diaphragmatic hernia (CDH) was the most common (eight cases). However, only one case of CDH was found at the 20-week ultrasound screening. Two cases were found after birth, one of which was an esophageal hiatal hernia, and the other was left-sided CDH without stomach prolapse into the thoracic cavity. Cardiovascular disease was the most common abnormality (50 cases), with VSD being the most common (16 cases), followed by vascular ring with the right aortic arch. Half of the cases with cardiovascular disease were detected at the 20-week ultrasound screening.

There were 13 cases with gastrointestinal abnormalities, most of which were detected at the 20-week ultrasound screening. Anal atresia was the most common (four cases), and all cases were detected after birth. All three cases of hepatomegaly were transient abnormal myelopoiesis associated with Down syndrome.

Of the 23 genitourinary abnormalities identified during the study period, pyelectasis was the most common, accounting for 11 cases. Nine (39%) genitourinary abnormalities were detected after the 20-week ultrasound screening. None of the four cases of ovarian cysts was detected at the 20-week ultrasound screening. Two cases of achondroplasia in skeletal dysplasia were not detected at the 20-week ultrasound screening. Of the nine cases of multiple malformations, five were detected at the 20-week ultrasound screening, including four cases of trisomy 18 and one case of vertebral, anal, cardiac, tracheoesophageal, renal, and limb abnormalities (VACTERL association).

## Discussion

The percentage of pregnant women with fetal structural abnormalities in this study was 1.3%, with 81.0% of them being found prenatally. More than half of the number of abnormal cases detected at the 20-week screening were newly detected at the 30-week screening. The earlier screening identified commonly detectable abnormalities, such as craniofacial and cardiac defects, whereas the later screening captured both abnormalities missed at 20 weeks and those that either emerged or became more evident in late gestation. The addition of 30-week ultrasound screening therefore provides enhanced diagnostic details in comparison to fetal ultrasound screening at 20 weeks.

Fetal structural abnormalities are generally found in 2–3% of all pregnancies [1], but our observed rate was lower than this reported range. Because this evaluation was conducted after the second trimester, cases with structural abnormalities (e.g., anencephaly, cystic hygroma) that are usually detected in the first trimester and cases with abnormalities found in prenatal genetic testing, including common trisomies, were excluded from this study. In addition, this study did not include congenital abnormalities found after the first postnatal week. In the third trimester of pregnancy, structural abnormalities were detected in 0.3% of all pregnancies in a recent systematic review [5]. The prenatal detection rate in the present study was 81%, which is similar to the approximately 70% seen in previous studies [1, 9, 10], although the sensitivity and specificity of ultrasound screening in detecting abnormalities depends on various factors, including the type of abnormality, quality of ultrasound examinations, and duration of postnatal follow-up [11].

The diseases identified at 20 weeks are common occurrences and are easily detected on ultrasonography. The prevalence of cleft lip and palate is higher in Japan than in other countries, ranging from 14.4 to 24.8 per 10,000 live births in a national survey [12]. Congenital heart disease (CHD) is the most common structural abnormality, the most frequent of which is VSD [13]. Vascular ring and heterotaxy syndromes are disorders that are likely to be detected in a screening program [14, 15]. Previous studies have noted that urological disorders can be easily detected at ultrasound screening [16]. Ventriculomegaly, which can be associated with chromosomal abnormalities, genetic syndromes, and neurodevelopmental delays, is frequently detected during prenatal ultrasound screenings [1].

Cardiovascular abnormalities identified at 30 weeks can be detected through ultrasound screening at approximately 20 weeks. However, poor cardiac visualization due to a lack of sonographer expertise, suboptimal fetal positioning, maternal obesity, or the presence of a cesarean abdominal scar can lead to failure to detect cardiac abnormalities. These unfavorable conditions can significantly affect the quality of ultrasound evaluations, particularly fetal echocardiography [17, 18]. In our cohort, most participants were of normal weight, with only a small proportion classified as obese. Therefore, while maternal obesity is known to negatively affect the quality of fetal imaging, particularly cardiac visualization, its influence on the detection rate in this study was likely minimal. Byrne et al. reported that an additional follow-up examination detected 45% of initially undetected fetal structural abnormalities [19].

Ovarian cysts, achondroplasia, and duodenal atresia, which were identified at the 30-week ultrasound, are not usually detected at the 20-week screening because their severity can progress during the prenatal period, and their symptoms often become apparent on ultrasound at approximately 30 weeks. Although some cases of pyelectasis and CDH can be detected during the 20-week ultrasound screening, some of them develop later in pregnancy, and disorder-specific findings may only become detectable during the 30-week ultrasound screening. Therefore, the 30-week screening complements the 20-week examination not only by detecting abnormalities that are missed due to limited visualization but also by identifying conditions that typically become apparent later in gestation. This dual role underscores the clinical value of incorporating a third-trimester structural assessment into routine prenatal care.

During the period between the 20- and 30-week screenings and after the 30-week screening, several abnormalities were detected. Some fetal abnormalities may not be detectable during the 20-week ultrasound screening owing to limited visualization, and certain conditions can develop later in gestation. Among these, conditions such as primary pleural effusion and CDH ideally require detection before 30 weeks to allow for timely intervention in severe cases [20, 21].

In the present study, 27 (19%) abnormalities were diagnosed after birth. These included VSD, anal atresia, spinal lipoma, cervical lymphangiectasia, CDH, and ventriculomegaly. Ventriculomegaly was caused by post-hemorrhagic hydrocephalus, believed to have resulted from hemorrhaging that occurred after the screening examination. Two cases of CDH diagnosed after birth were mild, with the stomach located in the abdomen, making a prenatal diagnosis challenging. In addition, we believe that spinal lipomas, low anal atresia, and intestinal rotation abnormalities are difficult to detect using the current prenatal ultrasound screening program. Cases discovered after birth were identified at a physical examination or became symptomatic by the time the newborn was discharged from the hospital. Thus, asymptomatic structural abnormalities that were not evident on routine physical examinations were likely missed. Some of these conditions, which are difficult to detect with prenatal screening examinations, may become symptomatic after birth, requiring new attempts at early detection, either prenatally or postnatally.

Some diseases detected using fetal ultrasound in the third trimester require immediate treatment after birth. For instance, duodenal atresia necessitates surgery shortly after delivery [22], and large ovarian cysts can sometimes undergo torsion during the perinatal period [23]. In addition, parents of fetuses diagnosed with achondroplasia may consider administering vosoritide to the child postnatally [24]. Although mild pyelectasis does not require immediate medication after birth, urological evaluation and follow-up are essential as some cases may progress in severity [25]. Since neonatal ultrasound is not routinely performed, fetal ultrasound can aid in early detection, ensuring timely check-ups to prevent future deterioration.

Several limitations associated with the present study warrant mention. First, it was a retrospective study conducted at a single center. Therefore, the results may not be applicable to other regions. Another limitation is inter-rater variability, as multiple obstetricians and sonographers were involved in conducting the screening examinations during the 7-year study period. While all examiners were board-certified or supervised by certified specialists, variations in skill and interpretation may have influenced detection rates.

## Conclusion

Our study showed that 81.0% of fetal structural abnormalities were prenatally detected. Of these, 42.3% were identified during the 20-week screening and 23.9% were identified during the 30-week screening. The frequency and types of detected abnormalities varied depending on the timing and type of ultrasound examination.

The addition of fetal ultrasound screening at 30 weeks provides enhanced diagnostic details in comparison to fetal ultrasound screening at 20 weeks by detecting abnormalities that are either missed or that become apparent later in gestation.

## Data Availability

The data supporting the findings of this study are available upon request from the corresponding author. The data are not publicly available because of privacy or ethical restrictions.

## References

[1] Edwards L, Hui L. First and second trimester screening for fetal structural anomalies. Semin Fetal Neonatal Med. 2018;23:102–11.29233624 10.1016/j.siny.2017.11.005

[2] Committee on Practice Bulletins—Obstetrics and the American Institute of Ultrasound in Medicine. Practice Bulletin No. 175: ultrasound in pregnancy. Obstet Gynecol. 2016;128:e241-e56. 10.1097/AOG.0000000000001815 27875472

[3] National Health Service (NHS), NHS fetal anomaly screening programme (FASP). Fetal anomaly screening programme handbook [Online]. NHS: London; 2015. https://www.gov.uk/government/publications/fetal-anomaly-screening-programme-handbook/overview. Accessed 2 Jan 2025.

[4] Salomon LJ, Alfirevic Z, Berghella V, et al. ISUOG Practice Guidelines (updated): performance of the routine mid-trimester fetal ultrasound scan. Ultrasound Obstet Gynecol. 2022;59:840–56.35592929 10.1002/uog.24888

[5] Drukker L, Bradburn E, Rodriguez GB, et al. How often do we identify fetal abnormalities during routine third-trimester ultrasound? A systematic review and meta-analysis. BJOG. 2021;128:259–69.32790134 10.1111/1471-0528.16468

[6] Itakura A, Shoji S, Shigeru A, et al. Guidelines for obstetrical practice in Japan: Japan Society of Obstetrics and Gynecology and Japan Association of Obstetricians and Gynecologists 2020 edition. J Obstet Gynaecol Res. 2023;49:5–53.36251613 10.1111/jog.15438

[7] Tokoro S, Koshida S, Tsuji S, et al. Insufficient antenatal identification of fetal growth restriction leading to intrauterine fetal death: a regional population-based study in Japan. J Matern Fetal Neonatal Med. 2023;36:2167075.36646445 10.1080/14767058.2023.2167075

[8] Khalil A, Sotiriadis A, D’Antonio F, et al. ISUOG Practice Guidelines: performance of third-trimester obstetric ultrasound scan. Ultrasound Obstet Gynecol. 2024;63:131–47.38166001 10.1002/uog.27538

[9] Romosan G, Henriksson E, Rylander A, et al. Diagnostic performance of routine ultrasound screening for fetal abnormalities in an unselected Swedish population in 2000-2005. Ultrasound Obstet Gynecol. 2009;34:526–33.19688769 10.1002/uog.6446

[10] Drukker L, Cavallaro A, Salim I, et al. How often do we incidentally find a fetal abnormality at the routine third-trimester growth scan? A population-based study. Am J Obstet Gynecol. 2020;223:919.e1-.e13. 10.1016/j.ajog.2020.05.052 32504567

[11] Whitworth M, Bricker L, Mullan C. Ultrasound for fetal assessment in early pregnancy. Cochrane Database Syst Rev. 2015;2015:Cd007058.26171896 10.1002/14651858.CD007058.pub3PMC6464767

[12] Tsuchiya S, Tsuchiya M, Momma H, et al. Association of cleft lip and palate on mother-to-infant bonding: a cross-sectional study in the Japan Environment and Children’s Study (JECS). BMC Pediatr. 2019;19:505.31862001 10.1186/s12887-019-1877-9PMC6923825

[13] Gordin Kopylov L, Dekel N, Maymon R, et al. Prenatally diagnosed isolated perimembranous ventricular septal defect: genetic and clinical implications. Prenat Diagn. 2022;42:461–8.35230708 10.1002/pd.6128PMC9313563

[14] Milligan I, Border W, Sachdeva R, et al. Contemporary outcomes in fetuses diagnosed with vascular rings. Pediatr Cardiol. 2024;45:1559–64.37354371 10.1007/s00246-023-03219-5

[15] Buca DIP, Khalil A, Rizzo G, et al. Outcome of prenatally diagnosed fetal heterotaxy: systematic review and meta-analysis. Ultrasound Obstet Gynecol. 2018;51:323–30.28603940 10.1002/uog.17546

[16] Dulgheroff FF, Peixoto AB, Petrini CG, et al. Fetal structural anomalies diagnosed during the first, second and third trimesters of pregnancy using ultrasonography: a retrospective cohort study. Sao Paulo Med J. 2019;137:391–400.31939566 10.1590/1516-3180.2019.026906082019PMC9745821

[17] Chaoui R. The four-chamber view: four reasons why it seems to fail in screening for cardiac abnormalities and suggestions to improve detection rate. Ultrasound Obstet Gynecol. 2003;22:3–10.12858294 10.1002/uog.187

[18] DeVore GR, Medearis AL, Bear MB, et al. Fetal echocardiography: factors that influence imaging of the fetal heart during the second trimester of pregnancy. J Ultrasound Med. 1993;12:659–63.8264018 10.7863/jum.1993.12.11.659

[19] Byrne JJ, Morgan JL, Twickler DM, et al. Utility of follow-up standard sonography for fetal anomaly detection. Am J Obstet Gynecol. 2020;222:615.e1-.e9. 10.1016/j.ajog.2020.01.001 31930994

[20] Wada S, Jwa SC, Yumoto Y, et al. The prognostic factors and outcomes of primary fetal hydrothorax with the effects of fetal intervention. Prenat Diagn. 2017;37:184–92.27977046 10.1002/pd.4989

[21] Deprest JA, Nicolaides KH, Benachi A, et al. Randomized trial of fetal surgery for severe left diaphragmatic hernia. N Engl J Med. 2021;385:107–18.34106556 10.1056/NEJMoa2027030PMC7613453

[22] Demirci O, Eriç Özdemir M, Kumru P, et al. Clinical significance of prenatal double bubble sign on perinatal outcome and literature review. J Matern Fetal Neonatal Med. 2022;35:1841–7.33455511 10.1080/14767058.2021.1874338

[23] Galinier P, Carfagna L, Juricic M, et al. Fetal ovarian cysts management and ovarian prognosis: a report of 82 cases. J Pediatr Surg. 2008;43:2004–9.18970932 10.1016/j.jpedsurg.2008.02.060

[24] Savarirayan R, Wilcox WR, Harmatz P, et al. Vosoritide therapy in children with achondroplasia aged 3-59 months: a multinational, randomised, double-blind, placebo-controlled, phase 2 trial. Lancet Child Adolesc Health. 2024;8:40–50.37984383 10.1016/S2352-4642(23)00265-1

[25] Sidhu G, Beyene J, Rosenblum ND. Outcome of isolated antenatal hydronephrosis: a systematic review and meta-analysis. Pediatr Nephrol. 2006;21:218–24.16362721 10.1007/s00467-005-2100-9

